# Bicarbonate buffer enhances functional sperm selection compared to Zwitterionic buffers in sperm preparation

**DOI:** 10.1038/s41598-026-44733-9

**Published:** 2026-03-18

**Authors:** Huidrom Yaiphaba Meitei, Dhakshanya Predheepan, Shubhashree Uppangala, Vani Lakshmi R, Guruprasad Kalthur, Stefan Schlatt, Satish Kumar Adiga

**Affiliations:** 1 Centre of Excellence in Clinical Embryology, Department of Reproductive Science, Kasturba Medical College, Manipal Academy of Higher Education, Manipal, 576104 India; 2 Division of Clinical Embryology, Department of Reproductive Science, Kasturba Medical College, Manipal Academy of Higher Education, Manipal, 576104 India; 3 Division of Reproductive Genetics, Department of Reproductive Science, Kasturba Medical College, Manipal Academy of Higher Education, Manipal, 576104 India; 4 Department of Data Science, Prasanna School of Public Health, Manipal Academy of Higher Education, Manipal, 576104 India; 5 Division of Reproductive Biology, Department of Reproductive Science, Kasturba Medical College, Manipal Academy of Higher Education, Manipal, 576104 India; 6 Centre of Reproductive Medicine and Andrology (CeRA), University of Münster, Münster, Germany; 7 Centre for Fertility Preservation, Department of Reproductive Science, Kasturba Medical College, Manipal Academy of Higher Education, Manipal, 576104 India

**Keywords:** Assisted reproductive techniques, Infertility, Spermatozoa, Sperm wash, Sperm recovery, Swim-up technique, Biological techniques, Biotechnology, Physiology, Urology

## Abstract

Sperm preparation media used in assisted reproductive technology (ART) laboratories typically contain single or combined buffers to maintain pH and support spermatozoa homeostasis. Concerns exist that zwitterionic buffers, such as 4-(2-hydroxyethyl)-1-piperazine ethane sulfonic acid (HEPES) and 3-(N-morpholino) propane sulfonic acid (MOPS), may negatively affect intrinsic cellular functions. Hence, this prospective study evaluated the impact of various buffers on sperm selection by swim up from fifty-four sub-fertile men, which were then divided into normozoospermic samples (*n* = 27) and non-normozoospermic (*n* = 27) cohorts. Each ejaculate was split into six aliquots, and swim-up was performed with media buffered by bicarbonate, HEPES, MOPS, bicarbonate + HEPES, or bicarbonate + MOPS. Post-swim-up spermatozoa were assessed for motility, morphology, kinematics, acrosome reaction, mitochondrial integrity, and DNA damage. Bicarbonate-buffered medium yielded spermatozoa with significantly higher motility and mitochondrial activity in both normozoospermic and non-normozoospermic samples (*p* < 0.001). In contrast, media containing HEPES or MOPS were associated with reduced motility (*p* < 0.001), compromised sperm kinematic parameters (*p* < 0.05), and diminished mitochondrial potential (*p* < 0.05). The study findings highlight the importance of bicarbonate buffer over zwitterionic buffers in sperm preparation media for the selection of better spermatozoa during ART procedures.

**Supplementary Information:**

The online version contains supplementary material available at 10.1038/s41598-026-44733-9.

## Introduction

The maintenance of pH is a crucial process during in vitro manipulation of spermatozoa, as extreme pH variation can irreversibly damage their fertilizing ability. Sperm motility and zona binding ability are influenced by the external pH (pHe) of the culture medium in which ejaculates are processed^1,2^. Chemically prepared buffers are the basis of sperm handling media. These are expected to protect sperm cells from pH variations and preserve sperm functionality.

Sperm cells are activated before fertilization by high bicarbonate levels, which facilitate lipoprotein-mediated cholesterol efflux, resulting in the modulation of sperm functional characteristics^3^. The bicarbonate buffer (HCO_3_^−^) is commonly used during the in vitro manipulation of spermatozoa. However, the buffering capacity of bicarbonate is carbon dioxide dependent; hence, it is challenging to maintain optimum pH while manipulating sperm cells outside the CO_2_ incubator^4,5^. Hence, zwitterionic buffers like HEPES (4-(2-hydroxyethyl)−1-piperazine ethane sulfonic acid) and MOPS (3-(N-morpholino) propanesulfonic acid) with a low concentration of bicarbonate are being used as the buffer system for gamete handling media^5^. HEPES contains a piperazine ring and is incorporated as a buffer system either in a free acid or conjugated form with various salts^5,7^. MOPS contains a morpholine ring and has a pKa ~ of 7.15 at 20 °C and is used as buffering biological systems or assays performed at a more neutral pH as free acid or sodium conjugates^5,7^. Since Zwitterionic buffers are independent of CO_2_^6–9^, using them in sperm handling medium allows the handling of samples outside CO_2_ incubators to maintain a stable pH^5^. On the other hand, the zwitterionic buffers do not support the physiological events in spermatozoa, such as capacitation, acrosome reaction, and hyperactivation as efficiently as bicarbonate^10,11^. Further, HEPES and MOPS during ICSI altered the oocyte transcriptome by increasing the transcripts of oxidative stress and lysosomal stress and decreasing chromosomal maintenance proteins^4^. It has been shown that spermatozoa selected using bicarbonate-based medium had higher curvilinear velocity compared to zwitterionic buffer-based medium^12^.

Owing to the zwitterionic buffer’s property of altering the cellular environment^4,5^, this study was undertaken to compare the specific effects of bicarbonate, HEPES, and MOPS buffers in selecting the functionally competent sperm from split human ejaculates.

## Methods

### Study subjects

Semen samples obtained from men referred to the University fertility clinic for infertility screening (*n* = 54) were categorised as normozoospermic (*n* = 27) and non-normozoospermic (*n* = 27) based on primary semen analysis. The fertility status of the female partner and the primary, secondary, or other investigations of the men were not performed. Patients were instructed to maintain 2–7 days of sexual abstinence before collecting an ejaculate for investigation. Semen analysis was performed as per the WHO fifth criteria^13^ except for sperm concentration analysis. The information regarding ejaculate characteristics is provided in Table 1. The study was conducted in accordance with the Declaration of Helsinki for conducting research involving humans, and it was approved by the Institutional Ethics Committees (IEC 520/2019). All the participants included in this study had provided written informed consent.

**Table 1:** Age and the semen characteristics of normozoospermic and non-normozoospermic cohorts.

	Normozoospermic (*n* = 27)	Non-normozoospermic (*n* = 27)
Patient age (in year)	36.93 ± 0.94	37.17 ± 0.69
Semen volume (mL)	2.81 ± 0.21	2.61 ± 0.18
Sperm concentration (10^6^/mL)	57.37 ± 4.02	12.29 ± 1.69
Total sperm number (10^6^/ejaculate)	152.11 ± 12.62	33.20 ± 7.52
Total motility (%)	62.16 ± 3.52	40.09 ± 3.62
Progressive motility (%)	35.52 ± 2.16	17.84 ± 1.96
Normal forms (%)	5.33 ± 0.36	5.46 ± 0.21

Values are expressed in mean ± SEM.

## Study design

Subjects were classified as having normal or abnormal sperm parameters according to the fifth edition of the World Health Organization criteria^13^. Each sample was split into six groups as follows:

1. Neat ejaculate (NE).
2. HCO_3_^-^: Sperm processing and swim-up step were performed using only bicarbonate-buffered medium containing 25 mM NaHCO_3_.
3. HEPES: Sperm processing and swim-up step were performed using only HEPES-buffered medium containing 21 mM HEPES and 4 mM NaHCO_3_.
4. HEPES+HCO_3_^-^: Sperm processing was performed in HEPES-buffered (21 mM HEPES and 4 mM NaHCO_3_) medium, and swim-up was performed in bicarbonate-buffered (25 mM NaHCO_3_) medium.
5. MOPS: Sperm processing and swim-up step were performed using only MOPS-buffered medium consisting of 21 mM MOPS and 4 mM NaHCO_3_.
6. MOPS+HCO_3_^-^: Sperm processing was performed in MOPS-buffered medium, and the swim-up step was performed in bicarbonate-buffered (25 mM NaHCO_3_) medium.

Sperm concentration was assessed using a Makler counting chamber (Sefi Medical Instruments Ltd., Israel), and motility was assessed manually in a wet preparation. Sperm morphology was assessed by the Shorr staining method^13^ using a brightfield microscope (Nikon Eclipse Ei). The sperm recovery rate was determined as previously described^14^.

## Preparation of buffered medium

Medium 199 (M199) from Sigma Aldrich, USA (Cat No. M5017) was used as a basal medium. NaHCO_3_ (Cat No. S5761, Sigma Aldrich, USA), HEPES (Cat No. H3375, Sigma Aldrich, USA) and MOPS (Cat No. M3183, Sigma Aldrich, USA) were added to the basal medium according to the experimental groups stated above. The medium pH was adjusted to 7.2–7.4 using 1 N sodium hydroxide (Cat No. 96311, SRL, India), and osmolality was adjusted to 280–290 mOsm/Kg using Gonotec^®^ Osmomat 3000. Before use, each batch of media was subjected to a sperm survival assay and used within three weeks.

## Sperm selection by Swim-up

The sperm selection by swim up was performed as described earlier^14^. Briefly, each ejaculate fraction was mixed with 1 mL of pre-warmed M199 medium, which had its matching buffering system supplemented with 0.1% bovine serum albumin (BSA). Centrifugation was done at 300 g for 8 min. The step was repeated two times to wash the pellet, and finally, the pellet was overlaid with 0.4 mL of M199 medium with the respective buffering system supplemented with 0.1% BSA. The tubes were incubated at 37 °C and 5% CO_2,_ tilted at a 45^°^ angle. After one hour, 0.2 mL of the supernatant containing sperm suspension was collected and evaluated further.

## Sperm kinematics assessment by CASA

8 µL of sperm suspension from each experimental group was taken on a pristine glass slide. Thirteen consecutive non-overlapping fields were captured, and sperm kinematics were assessed in 500 sperm cells at 37 °C using phase contrast microscope (UB200i) integrated with Computer Assisted Semen Analysis CASA software (Integrated Semen Analysis System; PROiSER, Spain) with a depth of 0.17 mm between the glass slide and the cover slip. Data on curvilinear velocity (VCL, µm/s), straight-line velocity (VSL, µm/s), average path velocity (APH, µm/s), amplitude of lateral displacement (ALH, µm), linearity (LIN, %), straightness (STR, %), wobble (WOB, %), and beat-cross frequency (BCF, Hz) were captured. The percentage of hyperactivated sperm cells was considered with VCL, LIN and ALH.

### Acrosome reaction

The acrosome reaction of the normozoospermic samples was performed in two approaches: spontaneous acrosome reaction and induced acrosome reaction. Assessment of spontaneous acrosome reaction was performed as described earlier^15^, with minor modifications^14^. 0.1 million spermatozoa were smeared on a clean coverslip after the swim-up preparation and were permeabilized with chilled 100% methanol. Alternatively, the ability to undergo induced acrosome reaction was assessed using calcium ionophore (A23187)-induced acrosome reaction (CIAR) assay as described earlier^14^ with minor modifications. 0.1 million sperm suspension was treated with or without 5 µM calcium ionophore (Cat. No. C7522, A23187, Sigma-Aldrich, USA) for 1 h at 37 °C and 5% CO_2_ using the experimental media. Post-incubation, washed cells with the respective experimental media were smeared on the coverslip and permeabilized in 100% cold methanol. The permeabilized cells from both approaches were stained with FITC-conjugated Pisum sativum agglutinin (FITC PSA; Cat. No. L0770, Sigma-Aldrich, USA) at a concentration of 25 µg/mL at room temperature for 30 min. Washed sperm cells were then counterstained with 7 µg/mL of propidium iodide (PI; P4170, Sigma-Aldrich, USA) and mounted on a clean slide using Dako mounting medium. A minimum of 500 spermatozoa were evaluated under a fluorescence microscope (Imager A1; Zeiss, Gottingen, Germany). Spermatozoa with no green acrosome cap were classified as acrosome-reacted spermatozoa, and the results were expressed in percentage^14^.

## Mitochondrial membrane potential

Sperm mitochondrial potential was assessed as described earlier^14,16^. Processed fraction containing approximately 0.5 million spermatozoa was incubated with 2 µM JC-1 (5,5,6,6’-tetrachloro-1,1’,3,3’-tetraethylbenzimi-dazoylcarbocyanine iodide; Cat. No. T3168, Molecular Probes, Life 151 Technologies, USA) at 37 °C and 5% CO_2_. After 20 min, sperm cells were washed and evaluated under the fluorescence microscope (Imager A1, Carl Zeiss, Germany). Spermatozoa showing bright red to yellow fluorescence in the region of the midpiece were classified to have functional mitochondria. A minimum of 200 sperm cells were assessed from each group to determine the percentage of sperm exhibiting intact mitochondria.

## Sperm chromatin dispersion (SCD) test

Sperm chromatin dispersion assay was carried out on processed spermatozoa as described earlier^17^ with minor modifications^18^. A mixture of 0.1 million spermatozoa and 1% low-melting agarose kept at 37 °C was layered on slides pre-coated with 0.65% of normal melting agarose and allowed to solidify. The slides were immersed in denaturation solution (0.08 N HCl), lysis solution 1 (0.4 M Tris, 20mM DTT, 1% SDS, 50 mM EDTA), and lysis solution 2 (0.4 M Tris, 2 M NaCl). The slides were then air-dried after being neutralized in Tris buffer (0.4 M Tris) and serially dehydrated in alcohol. Sperm chromatin integrity was assessed under a fluorescent microscope (Imager-A1; Zeiss, Gottingen, Germany) after staining the cells with ethidium bromide (7 µg/mL). The large halo spermatozoa (without any DNA damage or normal) produce halos with a thickness equal to or greater than the length of the minor diameter of the core, whereas small halo spermatozoa produce halos with a thickness equal to or smaller than 1/3 the diameter of the minor diameter of the core^17,19^. A total of 500 cells were assessed, and the percentage of spermatozoa with damaged DNA was calculated by counting spermatozoa with either a small or no halo. The percentage of spermatozoa with severely damaged DNA was calculated by counting spermatozoa with no halo.

### Statistical analysis

All data are presented as the mean and standard error of the mean (Mean ± SEM). Repeated measures ANOVA were used to observe differences in the parameters across the various buffers. Subsequently, Holm’s post hoc test was used to observe the role of bicarbonate buffer in HEPES and MOPS, respectively. Jamovi (version 2.3) was used for the statistical tests, and the graph was plotted by GraphPad Prism 8.0.1 (GraphPad Prism Software, CA, USA). The level of significance was set at 5% for the entire study.

## Results

### Bicarbonate buffer was more efficient in the selection of motile spermatozoa than Zwitterionic buffers

**Fig. 1:**
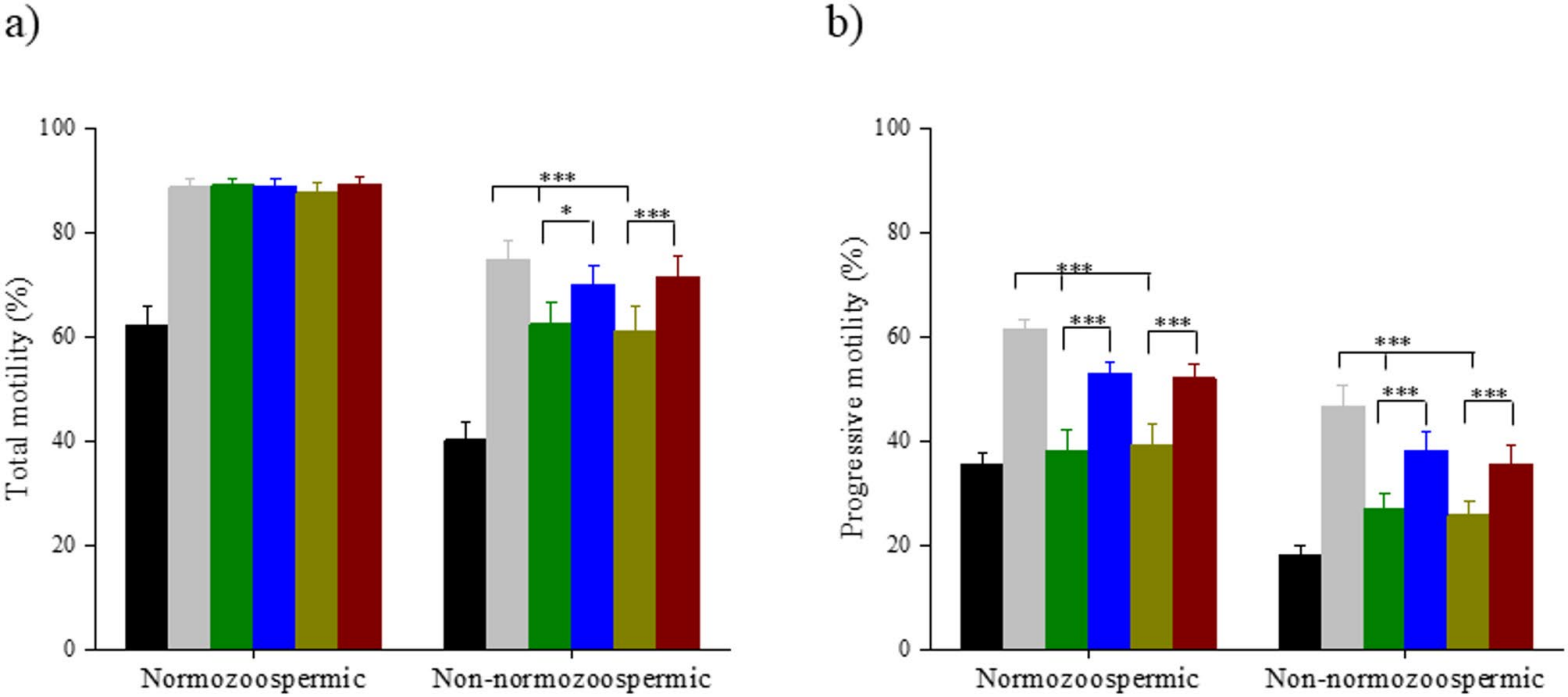
Influence of different buffers on sperm motility of normozoospermic (*n* = 27) and non-normozoospermic samples (*n* = 27). (**a**) Total motility and (**b**) Progressive motility. Repeated measures ANOVA was used for the comparison across the various buffers, and Holm’s post hoc test was used to observe the role of bicarbonate buffer in HEPES and MOPS. Values are expressed in mean ± SEM. Statistical significance was determined by *p* < 0.001(***) and *p* < 0.05 (*). Neat ejaculate (NE) (black); HCO_3_^−^ (grey); HEPES (green); HEPES+HCO_3_^−^(blue); MOPS (mustard); MOPS+HCO_3_^−^ (maroon).

The total sperm motility in the processed fraction was comparable across the groups in the normozoospermic ejaculates (Fig. 1A). In contrast, sperm selected from non-normozoospermic ejaculates using HCO_3_^−^ buffered medium had significantly higher total motility compared to HEPES (*p* < 0.001) and MOPS (*p* < 0.001). On the other hand, the combined use of HEPES/MOPS and HCO_3_^−^ buffered media resulted in a significant improvement in the total motility (HEPES+HCO_3_^−^; *p* < 0.05, MOPS+HCO_3_^−^; *p* < 0.001) (Fig. 1A).

Progressive sperm motility was approximately two-fold higher in the HCO_3_^−^ group in comparison to the HEPES and MOPS buffer groups in the normozoospermic cohort (*p* < 0.001). HEPES and MOPS alone failed to improve progressive motility in the processed fraction; however, when HCO_3_^−^ buffered medium was used for swim-up, a significant improvement in the progressive motility was observed (*p* < 0.001) (Fig. 1B). Similarly, in non-normozoospermic cohort, HCO_3_^−^ alone was efficient in selection of progressively motile spermatozoa in comparison to HEPES and MOPS (*p* < 0.001) whereas, HEPES+HCO_3_^−^ and MOPS+HCO_3_^−^ improved the progressive motility significantly (*p* < 0.001) in comparison with HEPES and MOPS alone (Fig. 1B).

**Fig. 2:**
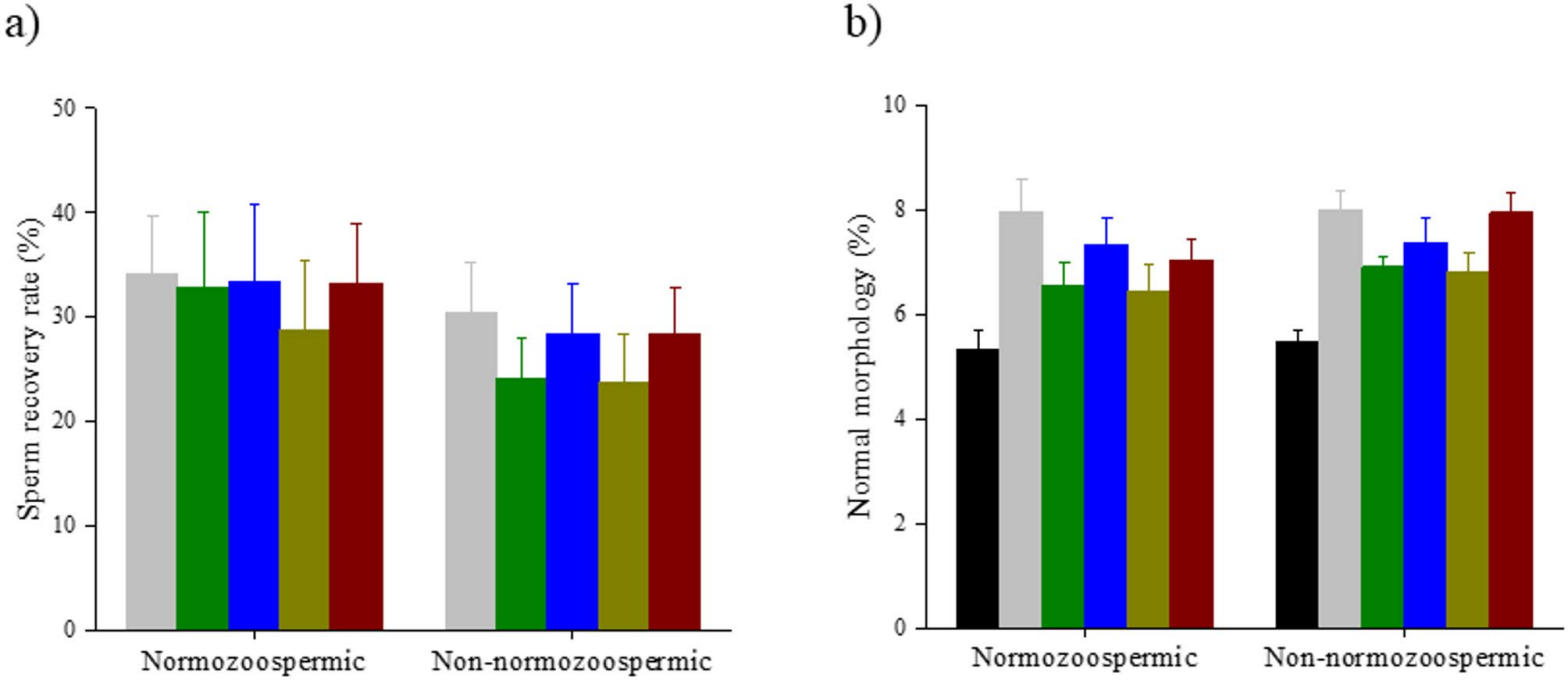
Influence of different buffers on sperm characteristics of normozoospermic (*n* = 27) and non-normozoospermic samples (*n* = 27). (**a**) Sperm recovery rate (**b**) Morphologically normal spermatozoa. Repeated measures ANOVA was used for the comparison across the various buffers and Holm’s post hoc test to observe the role of bicarbonate buffer in HEPES and MOPS. Values are expressed in mean ± SEM. Statistical significance was determined by *p* < 0.001(***) and *p* < 0.05 (*). Neat ejaculate (NE) (black); HCO_3_^−^ (grey); HEPES (green); HEPES+HCO_3_^−^(blue); MOPS (mustard); MOPS+HCO_3_^−^ (maroon).

The sperm recovery rate and the proportion of morphologically normal spermatozoa recovered from various experimental groups were comparable in both normozoospermic and non-normozoospermic cohorts (Fig. 2A and B).

When sperm kinematics of the normozoospermic fractions selected by swim up were compared across different study groups, the average curvilinear velocity (VCL, µM/s) and average path velocity (VAP, µM/s) were higher in HCO_3_^−^ compared to HEPES alone (*p* < 0.05, Table 2). Moreover, VCL and VAP improved significantly when combining HEPES with HCO_3_^−^. Similarly, the average curvilinear velocity (VCL, µM/s) was higher in HCO_3_^−^ compared to HEPES (*p* < 0.05) and MOPS (*p* < 0.05) in the non-normozoospermic cohort (Table 3). Importantly, the incidence of spermatozoa with wobble was significantly lower in HCO_3_^−^ buffered medium compared to HEPES (*p* < 0.05) in the non-normozoospermic cohort (Table 3). Other sperm kinematic parameters, such as Amplitude of Lateral Displacement (ALH), Linearity (LIN), Straightness (STR), and Beat-Cross Frequency (BCF), did not show any significant variations across the study groups. Overall, the percentage of hyperactivated sperm cells with sperm movement parameter VCL was higher in the HCO_3_^−^ group, while LIN and ALH were comparable across the study groups in both normozoospermic and non-normozoospermic samples.

**Table 2:** Sperm kinematics assessment by computer-aided sperm analysis (CASA) for normozoospermic samples (*n* = 27).

	HCO_3_^−a^	HEPES^b^	HEPES+HCO_3_^−c^	MOPS^d^	MOPS+HCO_3_^−e^	*p* < 0.05
Curvilinear velocity (VCL; µm/s)	65.30 ± 3.16	55.70 ± 3.77	66.10 ± 4.20	57.00 ± 4.04	64.50 ± 4.35	a vs. b; a vs. d b vs. c
Straight-line velocity (VSL; µm/s)	16.80 ± 1.29	17.90 ± 1.87	17.20 ± 1.38	16.70 ± 1.43	16.50 ± 1.06	NS
Average path velocity (VAP; µm/s)	33.50 ± 1.21	29.10 ± 2.12	34.10 ± 1.79	30.80 ± 1.74	34.80 ± 1.76	a vs. b; b vs. c
Amplitude of lateral displacement (ALH; µm)	2.10 ± 0.23	1.80 ± 0.20	2.10 ± 0.23	1.80 ± 0.20	1.80 ± 0.24	NS
Linearity (LIN; %)	25.70 ± 1.10	30.30 ± 2.09	26.70 ± 1.46	29.00 ± 0.94	26.00 ± 1.92	NS
Straightness (STR; %)	50.00 ± 2.66	54.40 ± 3.07	51.10 ± 2.81	53.40 ± 2.20	48.60 ± 2.57	NS
Wobble (WOB; %)	51.80 ± 1.53	54.90 ± 1.66	52.80 ± 1.63	54.60 ± 1.21	53.50 ± 2.15	NS
Beat-cross frequency (BCF; Hz)	6.60 ± 0.40	6.50 ± 0.63	6.50 ± 0.37	6.10 ± 0.72	6.40 ± 0.45	NS

Repeated measures ANOVA was used for the comparison across the various buffers and Holm’s post hoc test to observe the role of bicarbonate buffer in HEPES and MOPS. Values are expressed in mean ± SEM; Statistical significance was determined at *p* < 0.05. NS: Not significant at 5% level of significance. a, b,c, d,e, f represents HCO_3_^−^, HEPES, HEPES+HCO_3_^−^, MOPS and MOPS+HCO_3_^−^, respectively.

**Table 3:** Sperm kinematics assessment by computer aided sperm analysis (CASA) for non-normozoospermic samples (*n* = 27).

	HCO_3_^−a^	HEPES^b^	HEPES+HCO_3_^−c^	MOPS^d^		MOPS+HCO_3_^−e^	*p* < 0.05
Curvilinear velocity (VCL; µm/s)	56.00 ± 2.85	42.90 ± 2.15	48.60 ± 4.41	44.20 ± 2.74		54.60 ± 3.18	a vs. b; a vs. d
Straight-line velocity (VSL; µm/s)	17.10 ± 0.91	15.10 ± 0.84	15.50 ± 1.14	15.50 ± 1.00		15.60 ± 1.17	NS
Average path velocity (VAP; µm/s)	28.40 ± 0.77	25.60 ± 1.04	26.20 ± 1.81	26.10 ± 0.92		29.20 ± 1.23	NS
Amplitude of lateral displacement (ALH; µm)	1.40 ± 0.16	1.10 ± 0.17	1.30 ± 0.26	1.10 ± 0.17		1.30 ± 0.21	NS
Linearity (LIN; %)	32.90 ± 1.47	36.50 ± 2.31	33.20 ± 2.88	37.30 ± 3.57		29.20 ± 2.00	NS
Straightness (STR; %)	59.80 ± 1.83	60.40 ± 3.13	59.30 ± 3.09	61.10 ± 3.78		53.40 ± 3.08	NS
Wobble (WOB; %)	55.20 ± 1.09	60.00 ± 1.39	55.10 ± 2.21	59.90 ± 2.40		54.00 ± 1.03	a vs. b
Beat-cross frequency (BCF; Hz)	4.40 ± 0.80	5.00 ± 0.57	4.60 ± 0.76	4.90 ± 0.84		4.80 ± 0.61	NS

Repeated measures ANOVA were used for the comparison across the various buffers, and Holm’s post hoc test to observe the role of bicarbonate buffer in HEPES and MOPS. Values are expressed in mean ± SEM; Statistical significance was determined at *p* < 0.05. NS: Not significant at 5% level of significance. a, b,c, d,e, f represents HCO_3_^−^, HEPES, HEPES+HCO_3_^−^, MOPS and MOPS+HCO_3_^−^, respectively.

### Bicarbonate buffer enhances ionophore-induced acrosome reaction in normozoospermic samples

The impact of various buffers on the acrosomal reaction was attempted with two approaches. Initially, spermatozoa from all the experimental media were evaluated for spontaneous acrosome reaction and compared with the neat ejaculate (NE). The percentage of spontaneous acrosome-reacted spermatozoa was significantly reduced in the experimental media in relation to NE (*p* < 0.001). Alternatively, the percentage of ionophore-induced acrosome-reacted spermatozoa was higher in the HCO_3_^−^ group (34.6 ± 2.1%) compared to NE (25.8 ± 1.6%) (*p* < 0.01). However, there were comparable ionophore-induced acrosome-reacted spermatozoa between the experimental media (Supplementary Fig. 1).

### Bicarbonate buffer enhances the selection of spermatozoa with active mitochondria

**Fig. 3:**
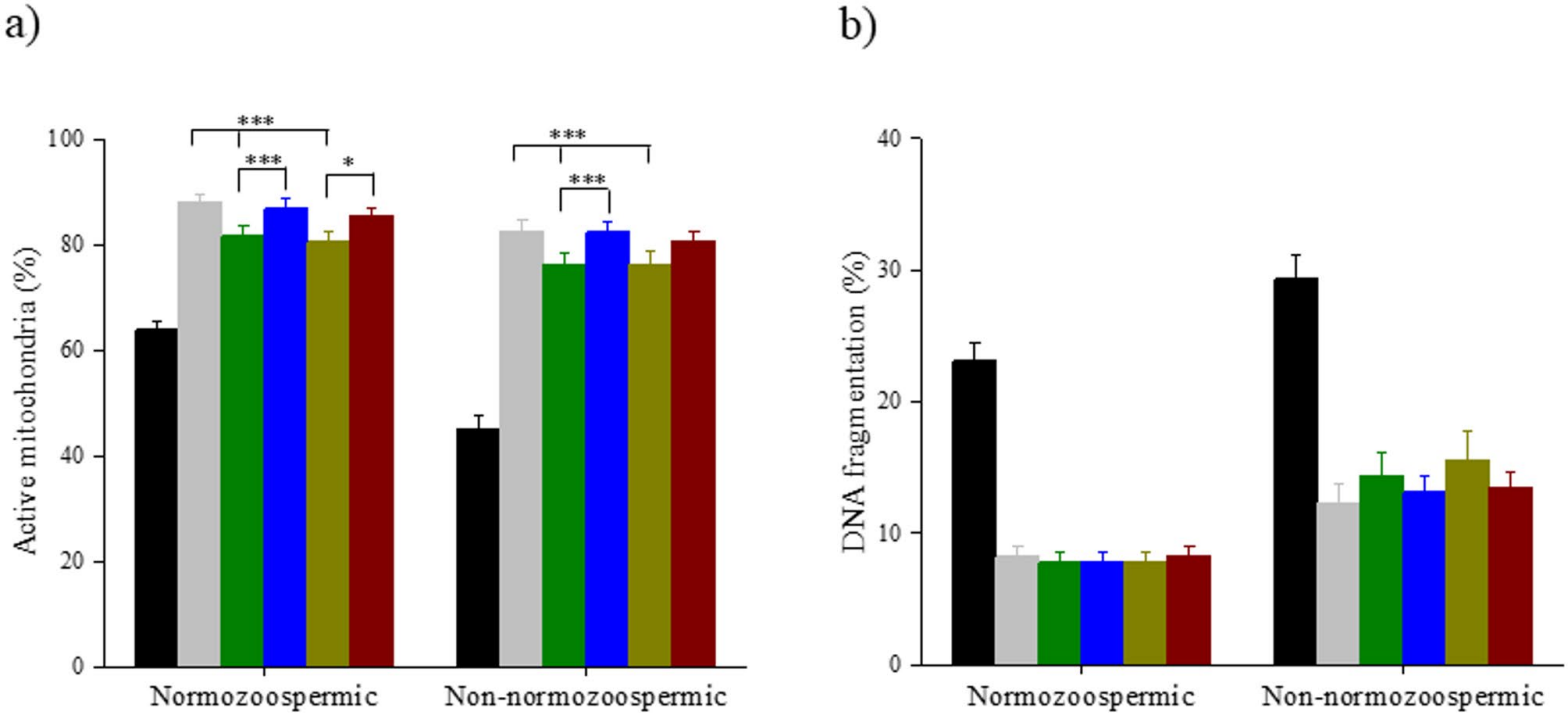
Influence of different buffers on sperm functional competence of normozoospermic (*n* = 27) and non-normozoospermic samples (*n* = 27). (**a**) Mitochondrial integrity (**b**) DNA fragmentation level. Repeated measures ANOVA was used for the comparison across the various buffers, and Holm’s post hoc test was used to observe the role of bicarbonate buffer in HEPES and MOPS. Values are expressed in mean ± SEM. Statistical significance was determined by *p* < 0.001(***) and *p* < 0.05 (*). Neat ejaculate (NE) (black); HCO_3_^−^ (grey); HEPES (green); HEPES+HCO_3_^−^(blue); MOPS (mustard); MOPS+HCO_3_^−^ (maroon).

In both normozoospermia and non-normozoospermic cohorts, the proportion of spermatozoa with active mitochondria was significantly higher in the HCO_3_^−^ fraction in comparison to HEPES and MOPS (*p* < 0.001). Combining HCO_3_^−^ with HEPES and MOPS improved the recovery of active mitochondria in normozoospermic ejaculates (*p* < 0.05), while combining HCO_3_^−^ with HEPES significantly improved the proportion of spermatozoa with active mitochondria in non-normozoospermic cohorts (*p* < 0.001) (Fig. 3A).

### Buffering system has not impacted the selection of DNA-intact spermatozoa

To evaluate the efficiency of a buffering system in selecting the DNA-intact spermatozoa, the SCD assay was performed on the experimental groups in the post-wash fraction. Since after swim up, most DNA-fragmented sperm were eliminated from the neat ejaculate (*p* < 0.01), the number of DNA-fragmented sperm was comparable in the post-wash fractions of all the study groups, both in normozoospermic and non-normozoospermic cohorts (Fig. 3B).

## Discussion

The observations made in this study suggest that the HCO_3_^−^ buffer is crucial in sperm preparation medium to select competent spermatozoa. On the other hand, Zwitterionic buffers alone cannot efficiently extract competent spermatozoa, both in normozoospermic and non-normozoospermic cohorts. To the best of our knowledge, this is the first study where the effect of specific buffer components was evaluated systematically on human sperm functional parameters in split ejaculates.

Minimizing exogenous stress in spermatozoa helps in maintaining the structural and functional competence and is a crucial component in attaining successful fertilisation in the IVF lab. One major stressor in the IVF lab while manipulating the ejaculates outside the incubator is pH, which is stabilized using buffers, such as HEPES and MOPS^5^. Sperm preparation medium normally contains HCO3^−^ buffer alone or in combination with HEPES or MOPS when sperm manipulation is done outside a CO_2_ environment. A combination of various pH buffers can be used to improve upon systems utilizing only individual buffers^5,20,21^. The acidic pH (6.6–6.8) in the male reproductive tract controls sperm motility, maturation and storage while the pH gradient in the female reproductive (4.2–7.5) controls alkalinization of the intracellular pH (pHi) of sperm cells^2,22^. The alkalinization of pHi of spermatozoa is essential for hyperactivation, capacitation, acrosome reaction and fertilization. The transport of HCO_3_^−^ and H^+^ is critical for the regulation of pHi^2,22^. There are a few concerns about the detrimental effects of buffers on the gamete and embryo quality^4,5,23–25^. Specifically, the direct exposure of ejaculates to chemically defined buffers during sperm preparation might impact the functional and genetic competence of spermatozoa. Therefore, closer examination of the effects of diverse buffers is required to develop optimal approaches.

Earlier studies have thoroughly assessed the impact of different sperm wash techniques and media composition on sperm recovery^24,26–28^. HCO_3_^−^ and serum albumin of defined media regulate sperm capacitation and Ca^2+^ exocytosis for acrosome reaction in either an independent or a dependent manner through different cascades of pathways^29–33^. MOPS buffer was found to be detrimental for the storage and freezing of boar spermatozoa, as it can alter the membrane integrity^5^. HEPES’s effectiveness with the storage of sperm from different species at different temperatures is well acknowledged^5^, though there are concerns about its use in ART^20,21^. Besides all these, HCO_3_^−^, MOPS, and HEPES-based media supplemented with serum albumin are being used for the sperm washing procedure in clinical practice^5^.

In the present study, the use of HEPES and/or MOPS buffers during wash and swim up in normozoospermic samples showed a significant reduction in motility (both total and progressive) compared to the HCO3^−^ group. This is possibly due to low bicarbonate availability in the medium (4mM) during manipulation, as the equilibrium in the conversion of CO_2_ and water into HCO_3_^−^ and H^+^ by the enzyme carbonic anhydrase (CA) controls pHi of sperm, hyperpolarization of plasma membrane, hyperactivation of motility, acrosomal exocytosis during capacitation and fertilization through a cascade of signalling pathways^33,34^. The partial improvement of sperm motility in combination with HEPES/MOPS with HCO_3_^−^ also highlights the importance of selection in the buffering system for sperm selection based on ART procedures^35^. It is also possible that HEPES/MOPS can interfere with many cellular functions that may inhibit the induction of motility during the sperm wash technique^5,20^. MOPS and HEPES have the capacity to interact with lipids, altering the membrane characteristics^5,36,37^; however, sperm morphology was unaffected across all study groups, though the impact of buffers on morphology is unlikely during sperm preparation.

Active mitochondria are important for sperm function^38–41^, and HEPES/MOPS can interfere with mitochondrial function by altering the electron transport pathway^5,37^ or producing oxygen-free radicals^42,43^. We observed that HEPES/MOPS-based medium, when used for the wash and swim-up procedure, negatively influenced the proportion of sperm with active mitochondria, indicating there might be interference in the equilibrium of H^+^ gradient affecting oxidative phosphorylation of mitochondria due to buffering systems^34^. However, DNA integrity in the processed fractions was not affected by various buffers.

The strength of the current study is that human ejaculate split fractions are used in all study groups, hence direct comparisons are possible. Further, media composition, albumin concentration and protocols were kept identical in all groups. This study also has limitations despite reporting the impact of buffering systems on sperm using split fractions from both normozoospermic and non-normozoospermic cohorts. The non-normozoospermic cohorts comprise oligozoospermic, asthenozoospermic and oligoasthenozoospermic samples. Hence, the generalizability of the results on sub-cohorts of non-normozoospermic samples may be limited. The sperm longevity assay was not performed for the extracted sperm cells. Furthermore, a limitation of the present study is the inability to perform additional functional competence assessments, such as protein tyrosine phosphorylation, mitochondrial ROS, mitochondrial DNA integrity, and genetic integrity tests, to elucidate the differential impact of buffering systems on sperm physiology and pre-fertilization events. The expression of several plasma membrane transporters necessary for the transport of HCO_3_^−^ and other ions was also not studied. Importantly, the fertilizing ability of the extracted spermatozoa was not assessed. This was due to the prospective clinical study wherein the semen samples were prioritized for patient treatment, and only residual samples were available for research purposes due to ethical restrictions. Furthermore, the use of a CO_2_-free incubator or a simple dry bath for the comparison between HEPES and MOPS will be the best option for a limited resource setting or a physician’s office for IUI programs. Further investigations with dedicated research samples and large cohorts should incorporate advanced functional and molecular assays to elucidate the underlying mechanisms of the differential response of spermatozoa exposed to various buffering systems.

## Conclusion

The observations support the importance of a physiological bicarbonate-based buffer for sperm preparation. It also highlights that bicarbonate buffer is preferable to attain sperm competency during swim-up procedures. Given that the zwitterionic buffers interfere with cellular function, our data provide experimental evidence that the selection of a buffering system for sperm preparation may be a determinant of sperm functional quality, emphasizing that the buffer selection should be based on clinical ART procedures.

## Supplementary Information

Below is the link to the electronic supplementary material.

Supplementary Material 1

Supplementary Material 2

## Data Availability

The datasets used and/or analysed during the current study are available from the corresponding author on reasonable request.
