# Supplementary figures and images for: Bicarbonate buffer enhances functional sperm selection compared to Zwitterionic buffers in sperm preparation
Source: Sci Rep. 2026 Mar 18;16:9332. doi: 10.1038/s41598-026-44733-9 (PMC13003109; doi:10.1038/s41598-026-44733-9)

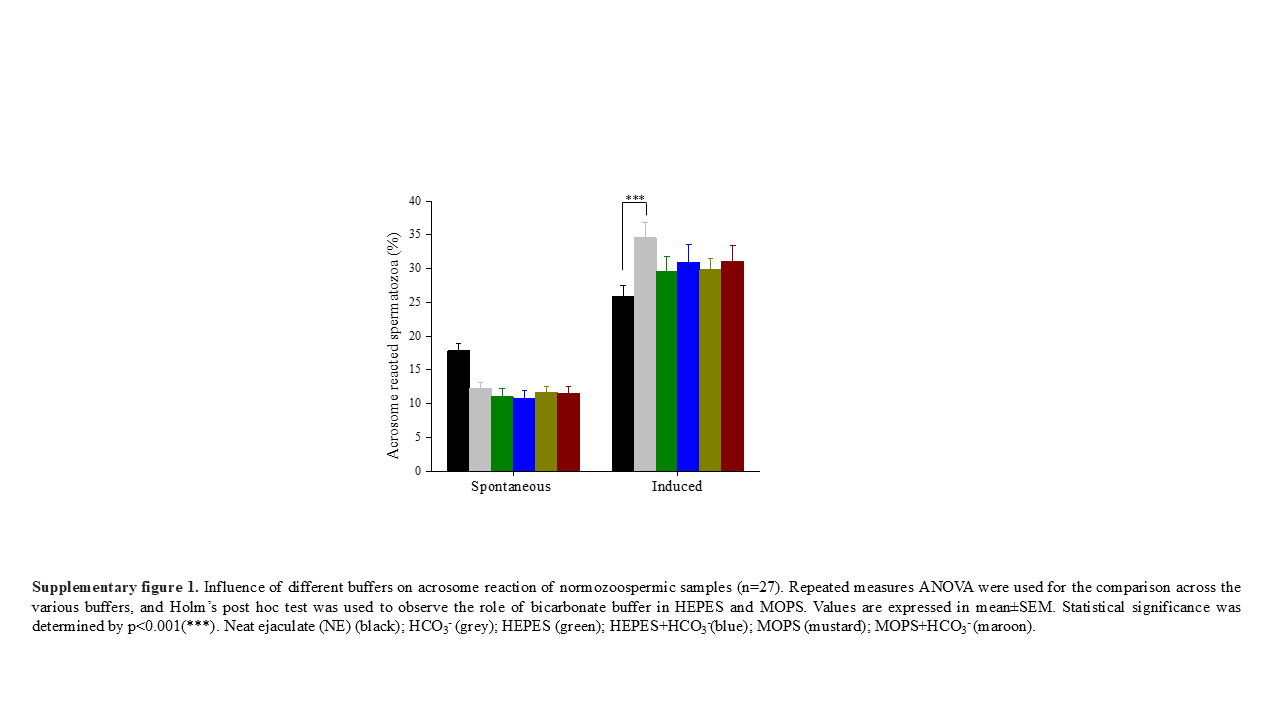

Supplement: Supplementary file 1 — Supplementary Material 1 [file 41598_2026_44733_MOESM1_ESM.tif]

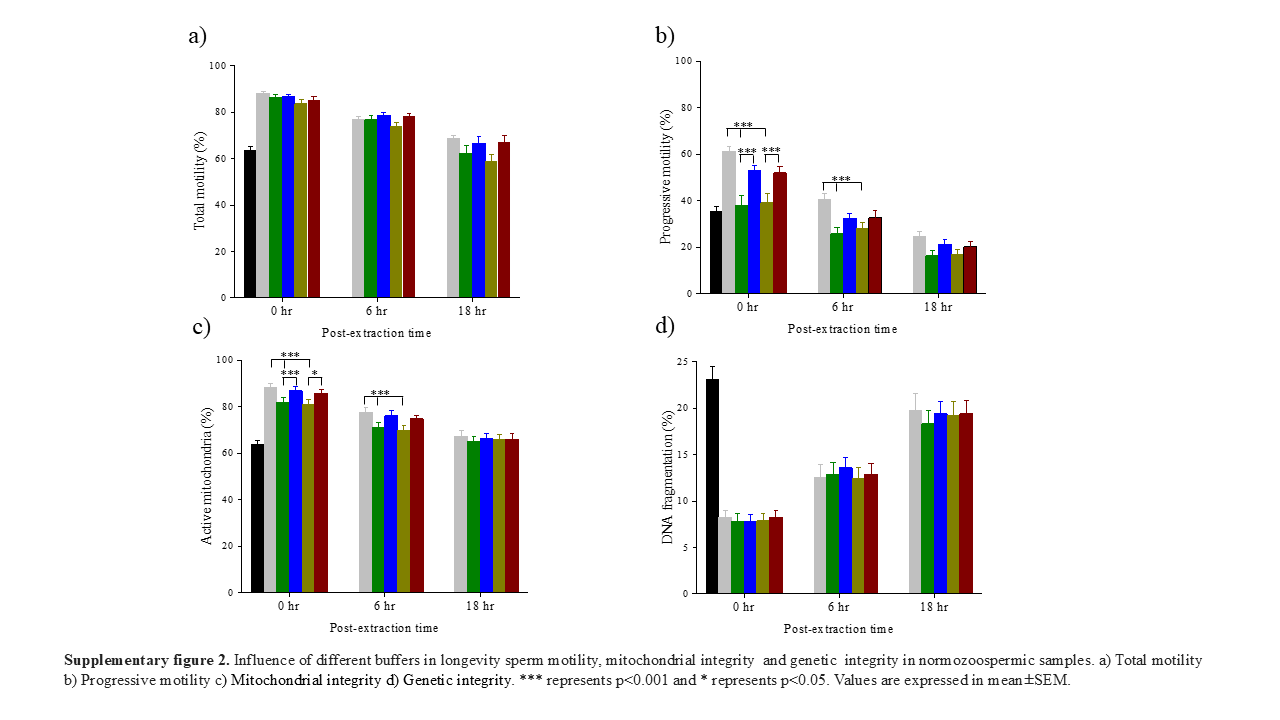

Supplement: Supplementary file 2 — Supplementary Material 2 [file 41598_2026_44733_MOESM2_ESM.tif]
